# Justification of CT practices across Europe: results of a survey of national competent authorities and radiology societies

**DOI:** 10.1186/s13244-022-01325-1

**Published:** 2022-11-22

**Authors:** Shane J. Foley, Ritva Bly, Adrian P. Brady, Steve Ebdon-Jackson, Alexandra Karoussou-Schreiner, Monika Hierath, Jacob Sosna, ESR EU-JUST-CT Project consortium, Boris Brkljačić

**Affiliations:** 1 A209 Health Sciences Centre, Radiography and Diagnostic Imaging, School of Medicine, University College Dublin, Dublin, Ireland; 2 STUK - Radiation and Nuclear Safety Authority, Vantaa, Finland; 3 Mercy University Hospital Cork and University College Cork, Cork, Ireland; 4 Medical Exposure Regulatory Infrastructure Team, CRCE, Public Health England, Chilton, Didcot, UK; 5 Radiation Protection Department, Health Directorate, Ministry of Health, Luxembourg City, Luxembourg; 6 European Society of Radiology, Vienna, Austria; 7 Hadassah Hebrew University Medical Center, Jerusalem, Israel; 8 School of Medicine, University of Zagreb, Zagreb, Croatia

**Keywords:** Computed tomography, Justification, Radiation protection, Survey

## Abstract

**Objectives:**

Published literature on justification of computed tomography (CT) examinations in Europe is sparse but demonstrates consistent sub-optimal application. As part of the EU initiated CT justification project, this work set out to capture CT justification practices across Europe.

**Methods:**

An electronic questionnaire consisting of mostly closed multiple-choice questions was distributed to national competent authorities and to presidents of European radiology societies in EU member states as well as Iceland, Norway, Switzerland, and the UK (*n* = 31).

**Results:**

Fifty-one results were received from 30 European countries. Just 47% (*n* = 24) stated that advance justification of individual CT examinations is performed by a medical practitioner. Radiologists alone mostly (*n* = 27, 53%) perform daily justification of CT referrals although this is a shared responsibility in many countries. Imaging referral guidelines are widely available although just 13% (*n* = 6) consider them in daily use. Four countries (Cyprus, Ireland, Sweden, UK) reported having them embedded within clinical decision support systems. Justification of new practices with CT is mostly regulated (77%) although three countries (Belgium, Iceland and Portugal) reported not having any national system in place for generic justification. Health screening with CT was reported by seven countries as part of approved screening programmes and by eight countries outside. When performed, CT justification audits were reported to improve CT justification rates.

**Conclusions:**

CT justification practices vary across Europe with less than 50% using advance justification and a minority having clinical decision support systems in place. CT for health screening purposes is not currently widely used in Europe.

**Supplementary Information:**

The online version contains supplementary material available at 10.1186/s13244-022-01325-1.

## Key points

- CT justification practices vary across Europe.
- Less than half of respondents reported advance justification of CT examinations.
- Imaging referral guidelines are widely available but not in daily use.
- CT for health screening is not widely used in Europe at present.

**Supplementary Information:**

The online version contains supplementary material available at 10.1186/s13244-022-01325-1.

## Background

Justification of medical exposures is one of the key tenets of radiation protection [1], which aims to ensure the benefit from radiological exposures always exceed any associated potential risks. This is particularly important in the modality of computed tomography (CT) which is increasingly being utilised and continues to be the largest contributor to population dose from medical exposures across many countries, despite its relative low frequency in comparison to other ionising radiation examinations [2–4]. Appropriate imaging referrals not only reduce the radiation exposure of the population, but importantly also save valuable healthcare resources. However, numerous publications point to a less than ideal level of justification in current practice with national audits reporting up to 39% [5–7] of CT examinations not being justified and even higher rates reported across smaller studies [8–11].

In 2021, the European Commission funded a three-year project on a coordinated action on improving justification of CT across Europe (acronym: EU-JUST-CT). The project performed under the auspices of the European Society of Radiology aims to collect up-to-date information about justification of CT examinations in Europe, to develop a common methodology for auditing justification of CT examinations, to carry out co-ordinated pilot audits of justification of CT examinations and to discuss the status of justification of CT examinations with the Member States and identify opportunities for further action. Work package 2 of the project was charged with conducting a survey to identify up-to-date information about justification of CT examinations in Europe and to collate data on previous audits on CT justification across European member states. This was done by surveying both the relevant radiation protection national competent authorities (NCAs) and the national radiological societies (NRS) across Europe. This article describes the survey and its results on behalf of the ESR EU-JUST-CT project consortium.

## Methodology

An ethical exemption was first obtained from the originating academic institution (LS-E-22-121-Foley) given the survey was being directed specifically to persons in public office or elected to professional societies speaking in a professional capacity and did not involve any sensitive topics. Although personal data (name and email address) were collected within the survey, these were used solely for the purpose of facilitating outreach to those participants whose submissions have been identified as requiring further clarification and/or context following review by the research team.

The research design centred around an electronic questionnaire created using a web-based platform (SurveyMonkey, Momentive, California). Initial survey design involved the entire EU-JUST-CT consortium team which was then piloted on members of both the project Advisory and the Steering groups (*n* = 10) as well as European Commission representatives, prior to being finalised. The survey contained 37 questions mostly multiple choice closed questions included for ease of completion, with open questions as appropriate. Questions were primarily split into five categories: respondent demographics, general justification and referral guidelines, justification of new practices, specific justification of CT examinations and previous audits (Additional file 1: Appendix 1).

The survey was distributed courtesy of the Heads of the European Radiological Protection Competent Authorities (HERCA) network to NCA contacts in each country and additionally it was circulated via the European Society of Radiology office to the Presidents of NRS of the European Union (EU) member states plus Iceland, Norway, Switzerland, and the UK (EU27+4). Survey distribution commenced in June 2021 and respondents were asked to complete it within a four-week period. Response rates were maximised by a system of regular reminders and personal follow-up as needed. Once the deadline was passed, survey responses were downloaded from the online software into Excel and data were first cleaned to remove incomplete responses or duplicate responses. Where duplicates were identified the most recent response was retained and the respondent emailed to confirm that this was appropriate. Results were then summarised using descriptive statistics and graphically displayed where appropriate.

## Results

A total of 47 responses was received by the initial July 2021 deadline. Following a three-week extension and direct contact with national contact persons, an additional nine responses were received by the August deadline. Two of these responses were incomplete with no answers completed beyond the demographic questions; these responses were discarded, along with three duplicate responses (most recent submission retained), leaving 51 completed responses for analysis (82% response rate). Responses were received from 30 countries, including 25 from NRS and 21 from NCAs with at least one response received for all countries except Liechtenstein (Table 1).

**Table 1:** List of respondents per country

Country	National radiological society	National competent authority
Austria	Austrian Radiological Society	–
Belgium	Belgian Society of Radiology	Federal Agency of Nuclear Control (FANC–AFCN)
Bulgaria	Bulgarian Association of Radiology	National Centre of Radiobiology and Radiation Protection
Croatia	Croatian Society of Radiology	–
Cyprus	–	Cyprus Regulatory Authority
Czech Rep	–	State Office for Nuclear Safety (SÚJB)
Denmark	Danish Society of Radiology	Danish Health Authority, Radiation Protection
Estonia	Estonian Society of Radiology	Environmental Board
Finland	Radiological Society of Finland	Radiation and Nuclear Safety Authority (STUK)
France	Société Française de Radiologie (SFR)	Autorité de Sûreté Nucléaire
Germany	Deutsche Röntgengesellschaft	Federal Office for Radiation Protection
Greece	Hellenic Radiological Society	Greek Atomic Energy Commission
Hungary	–	National Public Health Centre
Iceland	Radiological Society of Iceland	Geislavarnir ríkisins—Icelandic Radiation Safety Authority
Ireland	Faculty of Radiologists, Royal College of Surgeons in Ireland	Health Information and Quality Authority (HIQA)
Italy	Italian Society of Medical and Interventional Radiology (SIRM)	–
Latvia	Riga East University Hospital	–
Lithuania	Lithuanian Radiologists’ Association	Radiation Protection Center
Luxembourg	–	Radiation Protection department, Ministry of Health
Malta	Maltese Association of Radiologists and Nuclear Medicine Physicians	–
Netherlands	Dutch Society of Radiology	–
Norway	Norwegian Society of Radiology	Norwegian Radiation and Nuclear Safety Authority
Poland	Polish Medical Society of Radiology	–
Portugal	Sociedade Portuguesa de Radiologia e Medicina Nuclear (SPRMN)	–
Romania	Romanian Society of Radiology	National Commission for Nuclear Activities Control
Slovakia	Slovak Radiological Society (SRS)	–
Slovenia	Slovenian Association of Radiology and University College Maribor	Slovenian Radiation Protection Administration
Spain	Spanish Society of Medical Radiology (SERAM)	–
Sweden:	Swedish Society of Radiology (SFMR)	Swedish Radiation Safety Authority
Switzerland	Swiss Radiological Society	Federal Office of Public Health
United Kingdom	The Royal College of Radiologists	Care Quality Commission (England)

### Justification and referral guidelines

Most respondents (*n* = 32, 63%) reported that guidelines are available on the implementation of regulatory requirements for justification of medical exposures and that roles and responsibilities of the referrer and the radiology practitioner for justification of medical imaging examinations are defined in national regulations (*n* = 38, 78%). Respondents who reported that guidelines were not available for justification of (CT) medical practices included NCAs in Cyprus, Denmark, Estonia, Greece, Hungary, Iceland, Lithuania, Romania, Slovenia and Sweden and additionally NRS in Austria, Belgium, Estonia, Greece, Portugal, Slovenia.

**Fig. 1:**
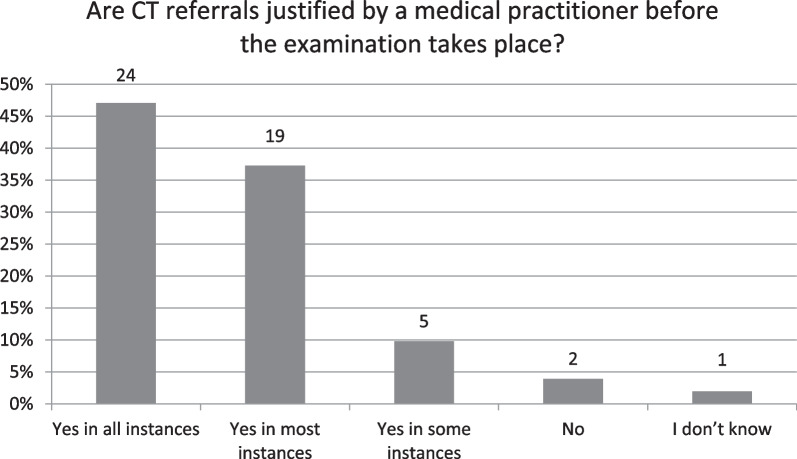
Are CT referrals justified by a medical practitioner before the examination takes place?

Specifically, regarding justification of CT examinations, when asked about whether justification of individual CT examinations was a legal requirement, just five respondents, namely the NRS from Austria, Belgium, Greece, Iceland and Portugal responded that it was not compulsory, with the majority answering that it was in all cases (*n* = 45, 88%). Similarly, just two respondents, the NRS from Belgium and Portugal, answered that CT referrals were not justified by a medical practitioner before the examination takes place with the majority indicating medical practitioner justification either in all cases (*n* = 24, 47%) or mostly (*n* = 19, 37%) (Fig. 1).

**Fig. 2:**
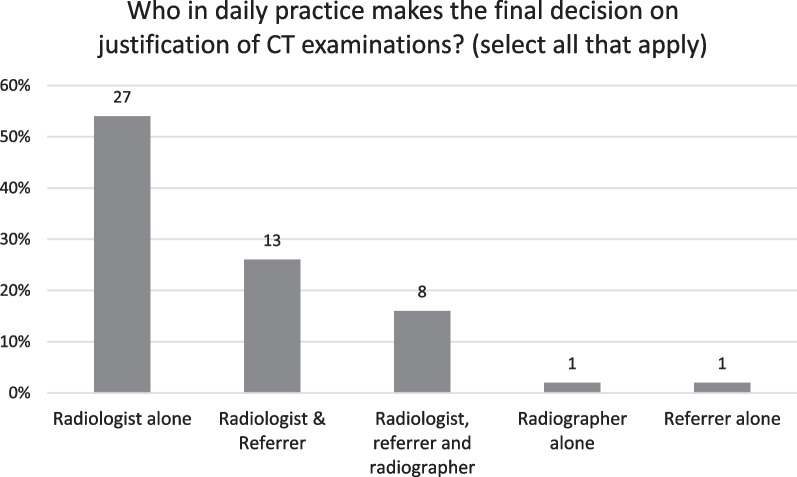
Who in daily practice makes the final decision on justification of CT examinations?

Radiologists alone predominantly (*n* = 27, 53%) made the final decision on justification of CT examinations daily (Fig. 2). However, this decision was reported by many countries to be a common effort between the radiologist and the referrer (*n* = 13, 26%), radiologist and the radiographer (*n* = 8, 16%) or all three professionals together (*n* = 2, 4%). One respondent (Slovakian NRS) stated that the referrer alone made the decision and another (Latvian NRS) that the radiographer alone did this. All respondents (*n* = 51, 100%) answered that the radiology practitioner has the legal right to change the CT referral to a more appropriate examination or to refuse a CT examination if the requested examination is inappropriate.

**Fig. 3:**
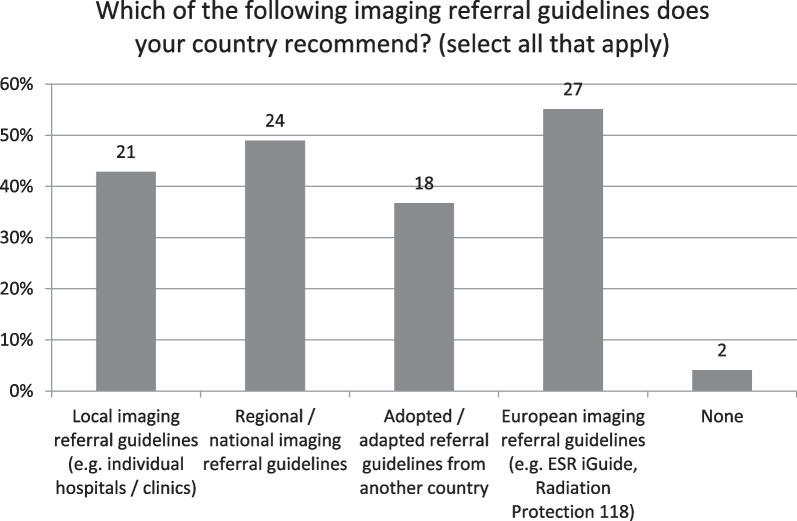
Which of the following imaging referral guidelines does your country recommend?

A range of imaging referral guidelines is available across countries, with European guidelines being most reported (*n* = 27, 55%) (Fig. 3). Just the Portuguese NRS and the Romanian NCA respondents reported that no referral guidelines were recommended nationally, although the Romanian NRS differed and reported that European guidelines were recommended. Respondents were asked about the availability of paediatric specific guidelines with just 24 respondents (49%) confirming their availability. Where guidelines were available, most respondents (*n* = 30, 61%) reported that they include information on radiation exposure. Guidelines were mostly available in electronic format across countries, with just 10 respondents (20%) reporting they were not, however only seven respondents (14%) from four countries stated that guidelines were incorporated into referral systems (clinical decision support), including NCAs from Ireland and Cyprus and both the NCA and NRS from Sweden and the UK.

**Fig. 4:**
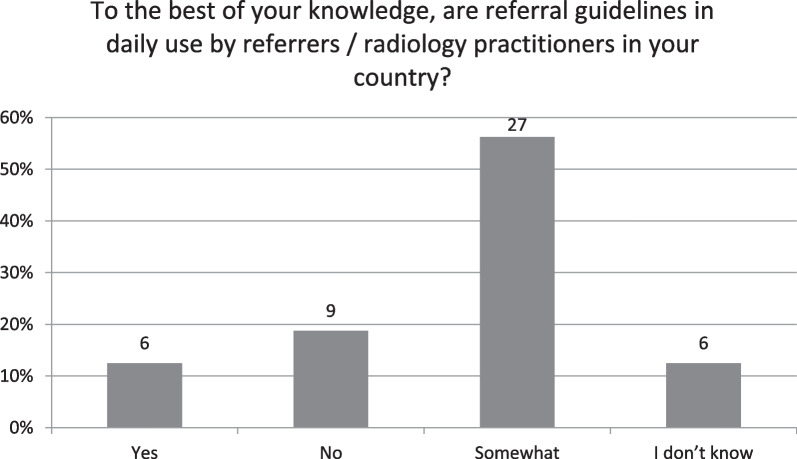
To the best of your knowledge, are referral guidelines in daily use by referrers/radiology practitioners in your country?

Just six respondents (13%) believed that referral guidelines were in daily use by referrers / radiology practitioners (Fig. 4). These included the Finnish and German NCA, Italian, Latvian, Norwegian, Romanian and Slovak NRS. Most (*n* = 27, 56%) stated they were somewhat used daily.

### Justification of new practices

**Fig. 5:**
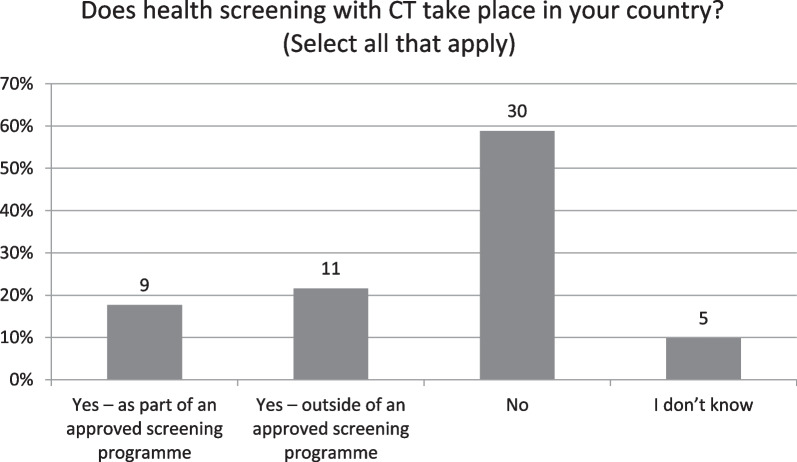
Responses to whether health screening with CT takes place in your country?

When asked about justification of new practices, most respondents (*n* = 39, 77%) replied that responsibilities for justification of new practices (with CT) were regulated, and that responsibility for initiating the process of justification of a new practice varied widely, ranging from health authorities (*n* = 5, 10%) to individual health practitioners (*n* = 6, 12%), professional societies (*n* = 6, 12%) and undertakings/licence holders (*n* = 13, 26%). Belgium, Iceland and Portugal respondents reported not having any national system in place for (generic) justification of new types of classes of CT practices [12]. The most common mechanism used for justification of new types of practices was via evidence-based procedures conducted by national societies (*n* = 26, 51%) or local hospital mechanisms (*n* = 25, 49%). Health screening with CT was reported by just 20 respondents (Fig. 5), with the majority (*n* = 30, 59%) not having such screening in place. Health screening with CT was reported by three NCAs (Bulgaria, Czech Republic, United Kingdom) and five NRS (France, Norway, Poland, Spain, United Kingdom) as part of an approved screening programme. Eleven respondents reported that CT screening occurred outside of an approved screening programme (NCA: Belgium, Bulgaria, Greece, Hungary, Switzerland and UK; NRS: Austria, Finland, Portugal, Spain, Switzerland). However, responses differed between the NCA and NRS in seven countries (Belgium, Bulgaria, Czech Republic, Finland, France, Greece, Norway) about whether screening with CT took place in their country. In four of these countries, the NRS responded in the negative (Belgium, Bulgaria, Czech Republic, Greece).

Just three respondents (NCA: Finland, UK; NRS: Germany) reported that guidelines are available from relevant medical societies and the NCA regarding the use of imaging for asymptomatic individuals outside of approved screening programmes, with another nine stating they are partly available (NCA: Switzerland; NRS: Austria, Bulgaria, Finland, Ireland, Norway, Poland, Romania, Spain). Eleven of seventeen respondents who reported using CT for health screening did have national regulations, with seven stating that such regulations included provisions about advertisement of CT health screening practices and just three (NCA: UK, NRS: Finland, Poland) that regulations allowed self-presenting of asymptomatic individuals.

### Previous audits

Respondents were asked whether any published audit/survey of the appropriateness of CT examinations had been carried out in their country in the past 10 years. Fifteen respondents from 11 countries (Croatia, Czech Republic, Estonia, Finland, France, Ireland, Italy, Luxembourg, Malta, Norway and Sweden), confirmed in the positive, while 10 did not know. Nine respondents provided links to these reported publications, three of which (Croatia [13], France [14], Ireland [15]) on further review were not specific to CT justification, while the Czech Republic, Estonian and Maltese audits were confirmed as unpublished audits. Respondents who reported a previous audit/survey of CT justification were also asked to identify any key outcomes/learnings from the audit, which are summarised in Table 2 below.

**Table 2:** Key outcomes from previous CT justification audits

Country	Key outcomes/results from CT appropriateness audits
Estonia	Follow-up audit showed significantly reduced CT numbers in the specific cohort (paediatrics)
Finland	The number of CT scans decreased significantly after the interventions and the level remained unchanged during the follow-up. Appropriateness improved significantly in CT scans already from 2005 to 2007
Luxembourg	CT appropriateness not satisfactory and collective efforts should be continued. The focus should be on general practitioners and on spinal CT examinations
Malta	Most audits showed poor adherence to guidelines in referral patterns
Norway	Large geographic variation in the use of CT and MR. Many examinations are already performed (mainly other places). CT is used when MR is more appropriate (due to availability and waiting lists)
Sweden	Generally high quality of the referrals. Radiologists often do not have the mandate to change the chosen modality without first talking to the referrer. The radiologists do not have direct contact routes with the referrers in primary care. The proportion of rejected referrals differs greatly between the X-ray clinics (from 0% to just over 8%). Formalised education in justification of medical exposures occurs in principle only in connection with Specialist Training programs for medical doctors and dentists

As anticipated, responses from within individual countries were not entirely consistent. When ‘I don’t know’ responses were disregarded it was noted that responses to specific questions varied when reported by the NRS or NCA, with a median of two different responses per country although this ranged from full agreement (zero differences: Denmark, Estonia) to seven different responses (Belgium).

## Discussion

The primary aim of the survey was to collect up-to-date information on justification of CT examinations in Europe, while additionally collating data on previous audits on CT justification across European member states. As responses were received from 30 countries, this survey presents valuable information on current CT justification practices across Europe, with perspectives from both the national competent authority (NCA) and national radiology society (NRS). Encouragingly, almost 90% of respondents replied that justification of individual CT examinations was a legal requirement in their country, with just five respondents, stating that it was not compulsory. This is despite Euratom/2013/59 Article 55 (2b) requiring all individual medical exposures to be justified in advance [16]. Additionally, just 47% then answered that all CT referrals were justified by a medical practitioner before the examination takes place (Fig. 1). Admittedly, just two respondents (Belgium and Portugal NRS) reported that CT referrals were not justified by a medical practitioner in advance, although this does not imply that the referral was not justified by anyone. While responses may have been influenced by differing interpretations of the question posed as to whether this related to real life practice or to the existence of regulation, it is clear that advance justification is not practically being performed for all CT examinations, given the volume of respondents (47%) who reported that advance justification was performed sometimes or mostly. Although a minority (*n* = 4) stated that referrals were justified at the point of referral, this could have significant implications for the appropriate use of CT for patients without radiology practitioner oversight, particularly when one considers the poor usage and knowledge of referral guidelines already cited by others [6, 8, 10, 17]. A previous HERCA report [18] following an inspection week in 2016 reported broadly similar findings, with as many as 26% of facilities not performing a satisfactory evaluation of the referral before the examinations were performed and even more not rejecting unjustified examinations (31%) or fully proving that the examinations were authorised by the radiological practitioner (35%). Given the current contribution of CT to population radiation doses [3, 4], it would be prudent to invest more resources into justification practices specifically in this modality, as substantial radiation dose savings could be achieved at a national level while also improving efficient use of healthcare resources.

Nearly all countries responded that referral guidelines were available nationally, with a range of guidelines being recommended, broadly similar to findings by Granata et al. [17] and the previous HERCA inspection [18], which reported that the sources of referral guidelines were national (58%), regional (15%) and/or local (28%). Results from this study however reported much higher usage of European guidelines (e.g. ESR i-Guide, RP118), likely to be due to the recent publication and promotion of same [19]. Similarly, it is clear from these survey results that referral guidelines are not in daily use by referrers and practitioners, with just 13% answering in the affirmative. Most (*n* = 27, 57%) stated they were somewhat used, again this is not dissimilar from the HERCA study which reported only ‘modest use of referral guidelines’, with guidelines assumed to be implemented in daily use by only 31% of the referrers and 48% of the radiological practitioners [18]. The reasons for same could be many fold, due to lack of availability or access to or familiarity with guidelines, culture and practice, work pressures or the possibility that either professional group (especially practitioners) may already be very familiar with the guidelines and not necessarily need to access them on a daily basis. Similar to a recent publication [17], less than half of respondents reported having paediatric specific guidelines available, despite these being a component of the most commonly used referral guidelines [19–21], similar results applied to the inclusion of information on radiation exposure. Both responses perhaps suggest a lack of familiarity with current imaging guidelines or alternatively could reflect the quite common (> 40%) use of local referral guidelines which are likely to vary in their content. Reassuringly here, results showed that almost all countries recommend imaging referral guidelines, most of which (80%) have them available in electronic format, although just seven respondents from four countries reported having guidelines incorporated into referral systems which would effectively compel their use and theoretically deliver better justification outcomes. This would not just assist with keeping both referrers and practitioners abreast of current evidence-based guidelines, but importantly could optimise patient pathways and save on unnecessary radiation exposure and wastage of precious healthcare resources.

When asked about health screening with CT, it is clear that CT is not widely used in this way across Europe, with only nine respondents from seven different countries stating it was part of an approved national screening programme, although a further eight countries reported CT was used outside of approved screening programmes (Fig. 5). Notably four of the countries (Belgium, Greece, Hungary, Portugal) using CT for screening outside of approved programmes did not report having guidelines available, which is likely to lead to heterogenous CT justification practices in this type of usage within and between countries. Regulation for the use of CT in health screening, where it does exist includes provision about advertisement in six countries (NCA: UK; NRS: Croatia, Finland, France, Norway, Poland, UK), with just three stating that self-presentation is permitted (NCA: UK; NRS: Finland, Poland). While this does not guarantee a CT examination will be performed on asymptomatic individuals, given the relative high doses associated with CT and the different benefit-risk ratio for such individuals, CT referrals should be carefully considered and should involve a medical practitioner in the decision-making regarding appropriateness of CT. Interestingly, the question regarding the existence of CT health screening generated the largest number of differing responses among respondents from the same countries, with respondents from seven countries providing responses different to those received from their national colleague(s). It is fully accepted that single respondents may not have the entire picture of clinical practices across an entire country, but surveys like this can be useful in initiating national conversations and sharing information among colleagues.

Regarding justification of new practices, encouragingly three quarters of respondents confirmed that responsibilities for new types or classes of practice involving CT were regulated and that responsibility for initiating the process varied between countries, with undertakings most commonly responsible. However, respondents from three countries (Belgium, Iceland and Portugal) reported not having any national system in place for such generic justification, which again can impact on the appropriate and evidence-based usage of CT. While mechanisms of assessment varied among respondents, it would seem sensible for countries to share results of their justification processes where possible, to minimise duplication of effort across Member States.

Questions on previous audits of CT justification proved helpful first in identifying further publications [22, 23] that were not included in the initial project literature review and also importantly highlighted upcoming planned audits by countries over the coming 24 months. Responses here largely tally with the results of the initial literature review, which demonstrated a dearth of publications on CT justification in Europe, again suggesting the necessity of further work in this domain. Additionally, the key outcomes (Table 2) identified by six countries who undertook such audits were very positive in terms of improving practices and reinforcing the benefits of such audit initiatives.

Regarding limitations, it was anticipated that different respondents from the same country might not always concur in their responses to questions, given their roles and particular perspectives. While there are benefits to this approach of including both the NCA and NRS perspectives, it also can lead to a lack of clarity in some areas, as responses from the same country varied. Further direct contact with respondents may have reduced these issues if time allowed. Regardless, this was an enlightening way to collect country-specific data and to encourage national dialogue to continue. It is proposed to circulate the survey report back to each of the respondents. Finally, as 14 countries had just one respondent (either NCA or NRS) it is acknowledged that the single responses received may not fully represent the national situation.

To summarise, these survey results provide up to date information about justification of CT examinations in 30 European countries from the perspectives of both the national competent authorities and the national radiology societies. The results highlight key areas for improvements, such as ensuring earlier involvement of the radiology practitioner in the process of justification of CT examinations, as less than half of respondents reported that CT referrals are always justified by a medical practitioner in advance of the examination taking place. While referral guidelines were reported as being available in almost all countries, it is clear that familiarity with and use of these is distinctly lacking and there exists a need to further encourage more regular use. Health services should strongly consider incorporating imaging referral guidelines into clinical decision support systems so that referrers will be required to review such guidelines before submitting a referral. CT for health screening purposes is not widely used at present in Europe either within or outside of approved screening programmes. More regular audits of CT justification are encouraged throughout all EU Member States to ensure this valuable imaging modality is used as efficiently and appropriately as possible.

## Supplementary Information

**Additional file 1: Appendix 1**. EU-JUST-CT Survey questions.

## Data Availability

The datasets used and/or analysed during the current study are available from the corresponding author on reasonable request.

## Abbreviations

CT: Computed tomography
EU: European Union
EU-JUST-CT: EU initiated CT justification project
HERCA: Heads of the European Radiological Protection Competent Authorities
NCAs: National competent authorities
NRS: National radiological societies
