# Supplementary material for: Justification of CT practices across Europe: results of a survey of national competent authorities and radiology societies
Source: Insights Imaging. 2022 Nov 22;13:177. doi: 10.1186/s13244-022-01325-1 (PMC9684387; doi:10.1186/s13244-022-01325-1)
Supplement: Supplementary file 1 — Additional file 1: Appendix 1. EU-JUST-CT Survey questions. [file 13244_2022_1325_MOESM1_ESM.pdf]

ELECTRONIC SUPPLEMENTARY MATERIAL

Justification of CT practices across Europe: results of a survey of national competent authorities and radiology societies

EC Tender Contract N° ENER/21/NUCL/SI2.844392

European co-ordinated action on improving justification of computed tomography

EU-JUST-CT

Background

The European Society of Radiology leads the European Commission funded EU-JUST-CT project, a European co-ordinated action on improving justification of computed tomography. The project will meet the following specific objectives:

a) Collect up-to-date information about justification of CT examinations in Europe.
b) Develop a common methodology for auditing justification of CT examinations.
c) Carry out co-ordinated pilot audits of justification of CT examinations.
d) Discuss the status of justification of CT examinations with the Member States and identify opportunities for further action.

Survey instructions

• Please fill in all text answers in English.

• The survey consists of a maximum of 37 questions and should take approximately 15-20 minutes to complete. A pdf copy however is also supplied in case you wish to consider the questions in advance of completing via the online submission.

• The closing date of the survey is July 30th, 2021.

• An asterisk '*' indicates a compulsory question

If you have any questions or difficulties, please contact XXX at XXX@myESR.org)

Thank you for your time and effort!

The EU-JUST-CT project has received funding from the European Commission under Service Contract N° ENER/21/NUCL/SI2.844392

Section 1: Demographics

*** . Permission to collect personal data:**

By checking this box, the participant confirms they have read and understand the above statement regarding collection of personal data and permit the collection and processing of their personal data as part of the EC-JUST-CT project. (check box)

- I agree

*** . Your country?** (drop down list)

*** . On behalf of what organisation are you responding?** (free text response)

*** . Title** (drop down list)

First Name (free text response)

*** . Last Name** (free text response)

*** . Email Address** (preferred email address for future queries / clarifications) (free text response)

Section 2: CT Justification

Relative to the application of justification in diagnostic CT in your country, please answer the following questions.

***. Are guidelines available on the implementation of regulatory requirements for justification of (CT) medical exposures?** (multiple choice – select one answer)

a) Yes
b) No
c) I don't know

*** . Is justification of individual CT examinations a legal requirement in your country?** (multiple choice – select one answer)

a) Yes, in all cases
b) Yes, only in the public system
c) Yes, only in the private system
d) No, it is not compulsory
e) I don't know

*** . Are CT referrals justified by a medical practitioner before the examination takes place?** (multiple choice – select one answer)

a) Yes in all instances
b) Yes in most instances
c) Yes in some instances
d) No
e) I don't know

*** At what time point are CT referrals deemed justified?** (multiple choice – select one answer)

a) At the point of referral
b) When the referral is scheduled
c) When the referral is vetted / reviewed by the radiology team
d) At the point of the CT examination, when the patient presents to CT

*** Who in daily practice makes the final decision on justification of CT examinations?** (select all that apply)

a) Radiology practitioner (e.g. radiologist)
b) Referrer
c) Practitioner and referrer together
d) Radiographer
e) Don't know
f) Other (please specify) __________________

*** Are responsibilities for justification of new types of practice (with CT) regulated?** (multiple choice – select one answer)

- ***a)*** Yes
- ***b)*** No

Please add any extra comments you have: ________________

*** Who has responsibility for initiating the process of justification of a new type of practice (with CT) (for example cardiac perfusion CT imaging)?** (multiple choice – select one answer)

- ***a)*** Health authority
- ***b)*** Radiation protection authority
- ***c)*** Professional society
- ***d)*** Undertaking / licence holder (e.g. hospital / imaging centre)
- ***e)*** Individual radiological practitioners
- ***f)*** Not applicable – no national system in place for level 2 justification
- ***g)*** Other (please specify)

*** What are the common mechanisms used for justification of new types of practice (with CT) (for example cardiac perfusion imaging)?** (select all that apply)

- ***a)*** Health Technology Assessment
- ***b)*** Evidence based procedures conducted by national societies of practitioners
- ***c)*** Local mechanisms at hospitals
- ***d)*** No mechanisms
- ***e)*** I don't know

*** Does health screening with CT take place in your country?** (select all that apply)

- ***a)*** Yes – as part of an approved screening programme
- ***b)*** Yes – outside of an approved screening programme
- ***c)*** No
- ***d)*** I don't know

*** . If health screening with CT takes place in your country, is it regulated?** (multiple choice – select one answer)

- ***a)*** Yes
- ***b)*** No

*** If health screening with CT is regulated, does his regulation include provisions about advertisement of CT health screening practices?** (multiple choice – select one answer)

- ***a)*** Yes
- ***b)*** No

c) Not applicable (screening with CT is not regulated)

*** If health screening with CT is regulated, does this regulation allow self-presenting of asymptomatic individuals to undergo opportunistic CT screening?** (multiple choice – select one answer)

a) Yes
b) No
c) Not applicable (screening with CT is not regulated)

*** Are guidelines available from relevant medical societies and the competent authority regarding the use of imaging for asymptomatic individuals outside of approved screening programmes?** (multiple choice – select one answer)

a) Yes (please specify) ________________
b) No
c) Somewhat / partly
d) I don't know
e) Not applicable – CT is not used for asymptomatic individuals (outside of approved programmes)

*** Which of the following imaging referral guidelines does your country recommend?** (select all that apply)

a) Local imaging referral guidelines (e.g. individual hospitals / clinics)
b) Regional / national imaging referral guidelines
c) Adopted / adapted referral guidelines from another country
d) European imaging referral guidelines (e.g. ESR iGuide, Radiation Protection 118)
e) None
f) Other (please specify)

*** Are paediatric specific imaging referral guidelines available in your country?** (multiple choice – select one answer)

a) Yes
b) No
c) I don't know

*** Where available, do referral guidelines take into consideration and include information on radiation exposure?** (multiple choice – select one answer)

a) Yes
b) No
c) I don't know

*** Are imaging referral guidelines available in electronic format (e.g. pdf, mobile app, internet) in your country?** (multiple choice – select one answer)

- ***a)*** Yes in all locations
- ***b)*** Yes in some locations
- ***c)*** No
- ***d)*** I don't know

*** In what format are imaging referral guidelines available?** (select all that apply)

- ***a)*** Hard-copy (paper based)
- ***b)*** Electronic – pdf documents available on internet / intranet
- ***c)*** Electronic – app for mobile devices
- ***d)*** Electronic – incorporated into referral systems (clinical decision support)

*** To the best of your knowledge, are referral guidelines in daily use by referrers / radiology practitioners in your country?** (multiple choice – select one answer)

- ***a)*** Yes
- ***b)*** No
- ***c)*** Somewhat
- ***d)*** I don't know

*** Are the roles and responsibilities of the referrer and the radiology practitioner for justification of medical imaging examinations defined in national regulations?** (multiple choice – select one answer)

- ***a)*** Yes
- ***b)*** No
- ***c)*** Partly

*** Does the radiology practitioner have the legal right to change the CT referral to a more appropriate examination if necessary, or even to refuse a CT examination if the referred examination is inappropriate?** (multiple choice – select one answer)

- ***a)*** Yes
- ***b)*** No
- ***c)*** I don't know

*** Has there been any published audit / survey of the appropriateness of CT examinations carried out in your country in the past 10 years?** (multiple choice – select one answer)

- ***a)*** Yes
- ***b)*** No
- ***c)*** I don't know

If there has been a published audit / survey of the appropriateness of CT examinations carried out in your country in the past 10 years, when was it? Please provide links to the associated results / report / publications if possible. (free text response)

If there has been a published audit / survey of the appropriateness of CT examinations carried out in your country in the past 10 years, was it a: (please select all that apply)

a) Regional audit
b) National audit
c) Local audit (e.g. single centre)

If yes, briefly describe the cohort of CT referrals audited (for example: Adult / paediatrics; specific body regions / all CT referrals; all CT referrals during a specific time period or CT referrals from a specific source (e.g. outpatients / emergency department / general practice); etc.) (free text response)

If there has been a published audit / survey of the appropriateness of CT examinations carried out in your country in the past 10 years, were there any key outcomes/results from the audit? (free text response)

*** Are there any audits / surveys of CT justification planned in your country for the next 24 month period?**

a) Yes (who is organising this audit / survey? Please add details of any planned audit if possible)
b) No
c) I don't know

Are there any on-going projects to establish referral guidelines or otherwise to improve the national situation on CT justification? (free text response)

Do you have any further comments relevant to this survey? (free text response)

Thank you!
